# Non-breeding European robins adjust their song in noisy environments

**DOI:** 10.1093/beheco/araf070

**Published:** 2025-06-12

**Authors:** Connor Proudfoot, William H J Norton

**Affiliations:** School of Biological Sciences, University of Leicester, University Rd, Leicester, LE1 7RH, UK; School of Biological Sciences, University of Leicester, University Rd, Leicester, LE1 7RH, UK

**Keywords:** bioacoustics, european robin, noise pollution, non-breeding song, urbanisation, vocalisations

## Abstract

Noise pollution is a global threat to biodiversity, significantly affecting acoustic communication in birds and other taxa. While European robins (*Erithacus rubecula*) adjust their songs in response to urban noise during the breeding season, little is known about song adjustments during the non-breeding season, when song plays a crucial role in survival by helping secure winter territories with adequate resources and shelter. To better understand the effect of noise on avian communication, we investigate whether robins modify their non-breeding song in noisy environments. We analyed the autumn songs of 25 robins exposed to varying background noise levels and found that robins increase the minimum frequency of their songs and extend phrase duration by singing fewer but longer syllables per phrase in noisy environments—adjustments that may mitigate acoustic masking. Our results provide valuable insights into the broader impact of urbanization on bird communication and highlight the need to consider non-breeding vocal behavior in conservation efforts. These findings underscore the year-round impact of noise pollution on birdsong, suggesting it affects various aspects of avian life history. However, it remains unclear whether these adjustments have evolutionary consequences for survival, as changes in key song parameters may affect how rivals interpret signals. Therefore, future studies should explore how vocal plasticity influences winter territory quality, predation rates, and individual survival.

## Teaser

Noise pollution disrupts bird communication, and we found that European robins change their songs in noisy environments in autumn. In the non-breeding season, song is crucial for defending winter territories needed for survival. Our study shows that in noisy areas, robins raise the minimum frequency of their songs and simplify their phrases. These adjustments may help them be heard but could also impact survival and competition with rivals.

## Introduction

Many animals use acoustic signals to control resources and interact with conspecifics, including both rivals and potential mates (Alexander 1961; Vergne et al. 2009; Baker et al. 2012; Conti et al. 2015; Knörnschild et al. 2017; Chen and Wiens 2020; Rodríguez et al. 2020; Indeck et al. 2021; Wijers et al. 2021). By broadcasting sound signals, individuals may reduce the energetic cost of competition and avoid injury, with conflicts fought out over distance (Smith and Price 1973). Because of their importance, both the information encoded within acoustic signals and their transmission can influence a signaler’s fitness, subjecting them to selective pressures (Nowicki and Searcy 2004). Among the most complex acoustic communication systems is that of passerine birds. Most species in this order have an innate ability to produce song which is shaped by hearing other songs during early development. They also have highly developed cortical regions that are specialized in producing vocalisations and compositional syntax (Beecher et al. 2007; Suzuki et al. 2018).

The selective pressures shaping avian vocal functions fluctuate with the seasons (Varpe 2017; Oosterveld and Frauendorf 2024). In the Western Palearctic, seasons are characterized by distinct changes in temperature and daylight, with cold, resource-scarce winters and warm, resource-abundant summers (Cramp and Simmons 1977). During the spring and summer breeding season, many songbirds sing to attract mates, deter rivals and guard resource-rich territories where they can raise their offspring. Because female songbirds often select a mate based on male song, sexual selection sculpts the composition of breeding songs (Catchpole 1987). In stonechats (*Saxicola rubicola*) and nightingales (*Luscinia megarhynchos*), both members of the old-world flycatcher family Muscicapidae, male song complexity correlates positively with brain size, health and paternal effort, and is selected for by females (Greig-Smith 1982; Bartsch et al. 2015). Song frequency also encodes meaningful information about the singer. Lower frequencies are perceived to be more threatening by same sex rivals but are more attractive to possible mates (Halfwerk et al. 2011). While reproduction dominates the breeding season, surviving winter to breed in the following season represents a major challenge. Non-breeding events significantly impact avian survival and fitness, both directly through mortality and indirectly by reducing body condition before the next breeding season (Brown and Sherry 2006; Frauendorf et al. 2025). Indeed, winter mortality in songbirds is so high that, in good years, fewer than half of individuals typically survive, driving species to evolve survival strategies (Adriaensen and Dhondt 1990; Sandercock and Jaramillo 2002). Many songbirds migrate to more temperate regions, while others remain resident and aggressively defend territories.

European robins (*Erithacus rubecula*) of both sexes use song to fiercely defend non-breeding territories, with song in winter more plaintive and lengthened relative to their breeding song (Lack 1953; Schwabl 1992; Schwabl and Kriner 2004; Collar 2020; Oosterveld and Frauendorf 2024). These territories are approximately 500 m², exclude mates and are dynamic both within and between seasons (Johnstone 2008). Several studies suggest non-breeding territoriality in robins is an adaptation to the increased selective pressure during winter imposed by avian predators like the Eurasian sparrowhawk (*Accipiter nisus*). In winter, safe cover is reduced and the availability of alternative prey for sparrowhawks is limited by migration (Cuadrado 1997; Dunn et al. 2003). Robins have a faster evasive response latency when exposed to a stuffed sparrowhawk in winter (Dunn et al. 2003), and can reduce their risk of predation by controlling territories that contain sufficient refuge and allow for safe foraging in resource-rich patches near cover (Cuadrado 1997). Territory-holding robins spend more than twice as much time foraging within two meters of shelter than non-territorial robins, which affords additional protection from potential threats (Cuadrado 1997; Schwabl and Kriner 2004).

Robins sing to establish and maintain safe territories, with autumn being a critical period when song elicits stronger behavioral responses and territorial interactions are most aggressive (Hoelzel 2008). At the same time, robins from more northerly breeding populations migrate and join resident British populations which further exacerbates competition for the best territories (Adriaensen and Dhondt 1990; Schwabl 1992; Cuadrado 1997; Oosterveld and Frauendorf 2024). Effective communication during the non-breeding season is critical for survival, as only individuals who survive this period can reproduce in the subsequent breeding season (Cuadrado 1997; Oden et al. 2015). Furthermore, male robins that sing more frequently in autumn and winter may gain a reproductive advantage in the breeding season (Hoelzel 2008). Therefore, factors that impair the transmission of acoustic signals may impact how robins control winter territories and reduce survival unless mitigated.

Many environmental factors affect how far long-distance acoustic signals transmit including the composition of vegetation, and ambient noise including noise pollution produced by humans (Ey and Fischer 2009). Human-induced rapid environmental change occurs too quickly for animals to adapt genetically, thereby imposing novel selective pressures on species. Therefore, behaviour offers essential flexibility, allowing species to adjust to these changes (Snell-Rood 2013). Noise pollution is a ubiquitous feature of cities that is generated as a byproduct of much human activity, most notably originating from transportation and industry (Nemeth and Brumm 2010), with high-intensity acute sounds exceeding 90 dB (Calixto et al. 2003). The information that vocalisations convey can be disrupted by noise pollution, a near-unavoidable outcome of human activity that threatens biodiversity in cities and beyond. As noise pollution is biased to low frequencies (less than 2000 Hz), it overlaps with the acoustic spectra of signals produced by birds, potentially interfering with their communication (Brumm and Slabbekoorn 2005; Hoelzel 2008; Slabbekoorn 2019). Specifically, the potency of low frequency signals is masked by anthropogenic noise (Halfwerk et al. 2011). Thus, because sending signals costs energy and has fitness consequences, many animals modulate their signaling behavior to maximize transmission fidelity and efficiency in noise (Blumstein and Turner 2005; Fuller et al. 2007; Ey and Fischer 2009).

Most adjustments made to rapid environmental change are underpinned by behavioral phenotypic plasticity (Slabbekoorn and den Boer-Visser 2006; Ey and Fischer 2009; Snell-Rood 2013; Kunc and Schmidt 2019, 2021). Plastic vocal adjustments to noise have consistently been found across species, from frogs and rodents to bats and birds, including European robins (Gross et al. 2010; Shier et al. 2012; McLaughlin and Kunc 2013; Shannon et al. 2015; Roca et al. 2016a; Kunc and Schmidt 2019, 2021). During the breeding season, many passerine species increase the pitch of their song to overcome acoustic masking (Slabbekoorn and Peet 2003; McLaughlin and Kunc 2013; Halfwerk et al. 2018; Zhan et al. 2021). Additionally, some species also modify their song structure and reduce complexity by singing fewer notes in noisy environments, however this may undermine sexual selection as more complex signals are indicative of male quality (Greig-Smith 1982; Montague et al. 2012; McLaughlin and Kunc 2013; McMullen et al. 2014; Bartsch et al. 2015) .

Although the effect of noise pollution on breeding season songs is now well-established, there are few studies on noise-induced vocal plasticity in non-sexually selected avian vocalisations (Roca et al. 2016b). Songbird behavior is affected by noise in the non-breeding season (Lehnardt et al. 2023), and Oden et al. (2015) found that American goldfinches (*Spinus tristis*) and black-capped chickadees (*Poecile atricapillus*) increase the minimum frequency of non-territorial non-breeding calls without changing the maximum frequency. Furthermore, the non-sexually selected begging calls of European starling (*Sturnus vulgaris*) also increase in pitch with noise (Dharmasiri et al. 2021) , while developing blue tit (*Cyanistes caeruleus*) vocalisations are also affected (Sierro et al. 2024). Despite this, studies examining the impact of noise pollution on the non-breeding songs of songbirds remain limited, partly because perennial singing is rare. Therefore, a comprehensive understanding of year-round vocal adjustments is essential to fully grasp how selection shapes song plasticity and influences a songbird’s life history.

This study explores the effect of noise pollution on the structure and frequency of European robin song in the non-breeding season, which functions to maintain a viable territory to survive the winter. Unlike song during the breeding season, non-breeding song is not under sexual selection, and it is therefore interesting to determine whether vocal adjustments persist and align with those reported in the breeding season. Historically, publications in this field reflect a bias towards reporting pitch components (especially minimum frequency) and a likely selective exclusion of non-significant results (Kunc and Schmidt 2021). Moreover, measures for song complexity are inconsistent across the literature, using one or more measures to score complexity (e.g., syllable rate, number of unique syllables or phrases, or derived complexity indices) (Boogert et al. 2008; McLaughlin and Kunc 2013). Therefore, we quantify multiple song components related to both frequency and structure, without derived complexity measures and model their relationship with background noise amplitude. Based on several previous studies on the European robin song in the breeding season (McLaughlin and Kunc 2013; Montague et al., 2013; McMullen et al. 2014), we expected robins in noisy environments to sing shorter, simpler phrases with a higher minimum frequency and fewer and longer syllables. These changes are expected because they may release the song from acoustic masking and improve signal transmission in noise. Lastly, given that high frequency elements are beyond the spectral overlap and avoid masking, we expected maximum song frequency to remain constant with noise (Montague et al., 2013).

## Methods

### Data collection

**Fig. 1.**
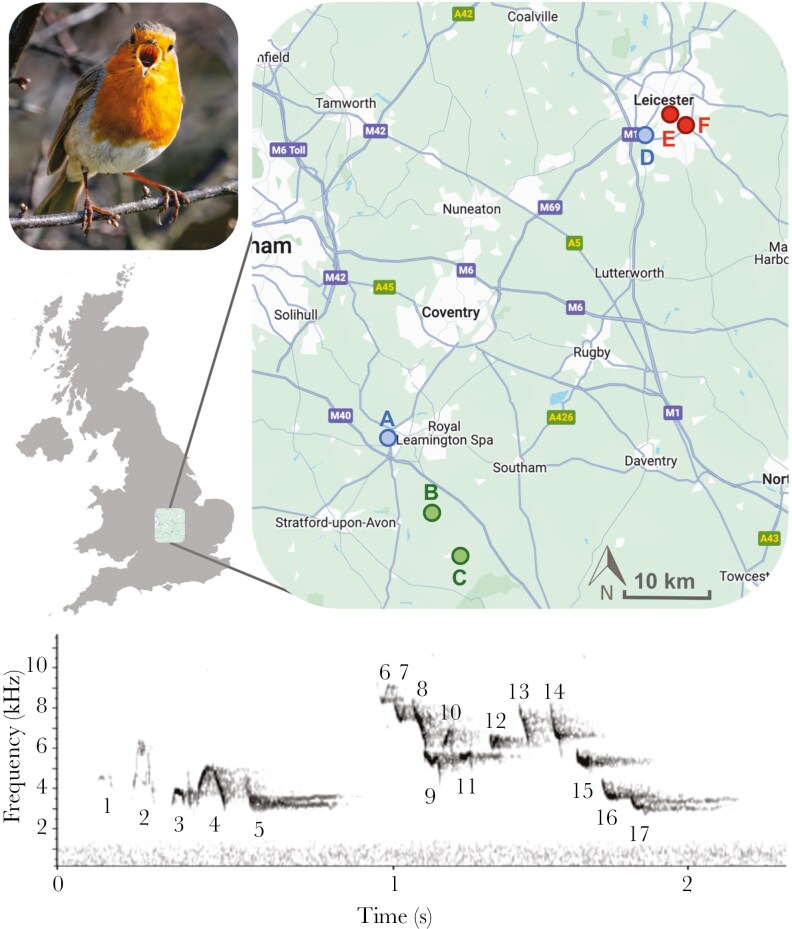
Six recording sites were sampled for European robin songs across Warwickshire and Leicestershire, England: (A) Warwick Racecourse, (B) Oakley Woods, (C) Lighthorne Village, (D) Aylestone Meadows Local Nature Reserve, (E) Victoria Park and (F) London Road, Leicester. Urban and rural areas are shaded in gray and green respectively and major roads are shown by dark gray lines (Map from Google Maps). Highly urban locations are highlighted in red and the rural locations in green. Peri-urban locations at the edges of urban and rural areas are shown in blue. A spectrogram of a typical European robin song phrase recorded in Oakley Wood, with a background noise level of 31 dB is shown below. This song contains 17 syllables (numbered 1 to 17) and has a duration of 2.2 s. The gray noise band visible below 1 kHz was produced by distant road traffic noise. Photo: Connor Proudfoot

This research was conducted on European robins recorded from October to December 2022 in Leicestershire and Warwickshire, England (Fig. 1). These months cover the onset of winter, a period when European robins are establishing their non-breeding territories (Hoelzel 2008; Oosterveld and Frauendorf 2024). A Marantz PMD661 MKIII solid state hard drive with a Sennheiser MKE600 directional microphone was used by a single recordist to collect song recordings (sampled at a frequency of 44.1 kHz and 16-bit amplitude resolution). Based on past studies and our preliminary recordings, we determined seven song parameters of interest—namely syllable count and duration, phrase rate and duration, minimum and maximum frequency, and the frequency range (bandwidth). After this period, data collection took place at six sites between 7:00 and 10:00 and was avoided during wind speeds greater than 15 km/h (Met Office UK: https://www.metoffice.gov.uk/) or precipitation to mitigate temporal and environmental variation in song (Schäfer et al. 2017). To avoid pseudo-replication by recording the same individual multiple times, London Road, Victoria Park, Lighthorne Village, and Warwick Racecourse were sampled once, whereas Oakley Wood was sampled twice and Aylestone Meadows was sampled three times due to their larger areas. Road traffic was the primary source of noise pollution at all sites and varied both spatially and temporally within and between locations. We surveyed the sites from paths and when a singing robin was found. We positioned ourselves 5 to 10 meters from the bird and waited for 60 s before recording to reduce the effect of approaching. Visitors were frequent at the locations, therefore the robins we studied were habituated to human presence. We recorded a total of 32 songs from adults of both sexes for their duration and in all cases, the song had already started before recording. Recording date, time, location, and weather conditions were noted and sound files were saved in.MP3 format for analysis.

Background noise was measured immediately following a recording when the focal individual had stopped singing. This was done using the Decibel X application on an iPhone 8, with default settings and A-weighting applied (McLaughlin and Kunc 2013; Erbe et al. 2022). We acknowledge the potential limitations of using a smartphone for measuring background noise but chose this method due to its availability and to test its applicability in bioacoustics studies. Previous work highlights the significant value of mobile crowd sensing and citizen science to large-scale urban ecology studies by leveraging the omnipresence of smartphones (Zipf et al. 2020). To ensure the reliability of measures, a detailed and consistent procedure was used. Noise amplitude was measured from the recording position by pointing the microphone north for 60 s and determining the average decibel level. This process was repeated with the microphone facing east, south, and west and the mean noise amplitude was calculated using the four directional measures.

### Analysis

European robin songs contain many short phrases composed of syllables (Hoelzel 2008). Syllables are unbroken units of vocalization that are temporally separated from adjacent syllables with specific inflections, durations, and frequencies (Hoelzel 2008; Suzuki et al. 2018). Many syllables placed in sequence form a phrase, which is separated from adjacent phrases by at least one second without vocalization. To analyze the robin songs, spectrograms were generated in Raven Pro (version 1.6.4, available at https://www.ravensoundsoftware.com/) (Lisa Yang Center for Conservation Bioacoustics 2024). Sound files were examined for quality. Seven recordings met our exclusion criteria and were not analyzed. Files were excluded from analysis if there were overlapping sounds that significantly obscured the target vocalisations (e.g., other bird vocalisations, wind noise, acute anthropogenic noise) (n = 2 songs excluded). Files were also excluded if the amplitude of the vocalization was too weak to produce a strong spectrogram signal (n = 2 songs excluded), if they were shorter than 120 s and contained fewer than 10 complete phrases (n = 3 songs excluded). Therefore, a total of 25 individual songs were analyzed. Each complete phrase in a song recording was coded, and for every phrase we scored four structural parameters (syllables per phrase, syllable duration, phrase rate and phrase duration) and three frequency parameters (minimum and maximum frequency, and frequency bandwidth). This sample size was similar that of previous work in this species (McLaughlin and Kunc 2013). Scoring was done by a single, trained observer blind to the background noise amplitude and location. The values used in data analysis were the parameter averages across all complete phrases, except for phrase rate which was calculated across the full recording (see supplemental data for more details).

Bayesian linear mixed models (BLMMs) examined the effect of background noise on several robin song features, run in RStudio (version 4.3.3) (R Core Team 2024). These models use the blme extension (Chung et al. 2013) of the R-package lme4 (Bates et al. 2015) to expand its functionality to include Bayesian inference. We employed this approach because BLMMs generate more robust estimates using Markov chain Monte Carlo methods to estimate the posterior distribution of model parameters (Chung et al. 2013). Estimates are provided with standard error.

To isolate the effect of background noise from potential location effects (including habitat type, degradation, and disturbance), a full-null model comparison was conducted. For each song parameter, a full model was fitted with mean-centered background noise amplitude (dB) as a fixed effect and recording locations as random effects with random intercepts, controlling for location-specific differences by allowing a separate intercept for each location. This accounts for any variation in song parameters across locations when estimating the effect of background noise. Null models for each parameter, including only locations as random effects, were also constructed. Model assumptions were checked using diagnostic plots and a Shapiro-Wilk test (shown in supplemental materials). To meet the assumptions of BLMMs, maximum frequency and frequency bandwidth were transformed using a square root reflection transformation, √ (*maxvalue*(*x*) + 1)—*x*, to address the left-skewness in the data, while phrase rate was square root transformed. A likelihood-ratio test (LRT) compared the fits of the full and null models with Akaike information criterion (AIC) and Akaike weights evaluating model support. Here, a lower AIC value identifies the most parsimonious model. Additionally, marginal R² and conditional R² values were calculated using the performance package (Lüdecke et al. 2021). The marginal R² represents the variance explained by the fixed effects (background noise) alone, while the conditional R² includes both fixed effects and random effects (location differences). The difference between the two reflects the variance explained by the location random effects, accounting for additional variation in song parameters not explained by the fixed effects. Data visualization was performed using ggplot2 (Wickham 2016) and patchwork (Pedersen 2024), with 95% confidence intervals calculated using ggeffects (Lüdecke 2018).

## Results

**Fig. 2.**
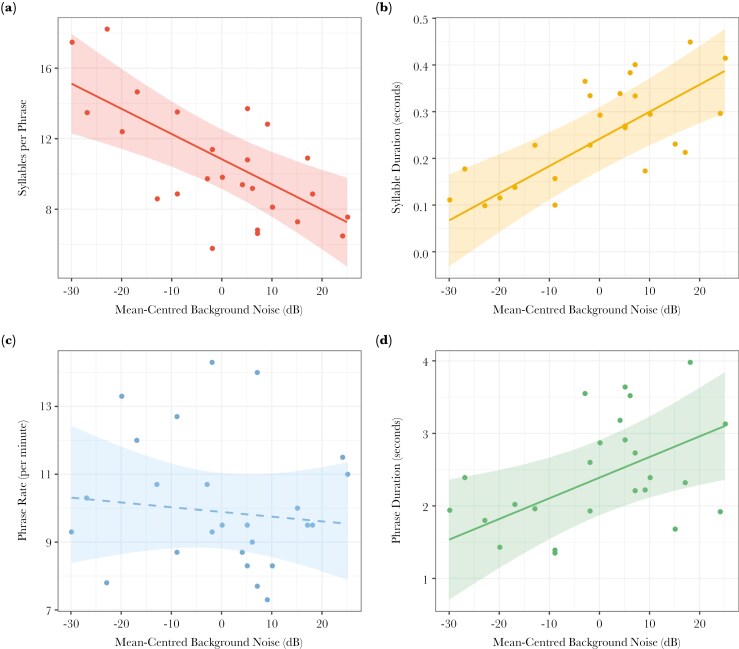
The effect of mean-centered background noise amplitude on four structural variables in European robin songs: (A) syllable rate, (B) syllable duration, (C) back-transformed phrase rate, and (D) phrase duration. Significant and non-significant relationships are indicated by solid and dashed lines, respectively. Dots represent mean individual song measures, and the shaded ribbons show the model-derived 0.95 confidence intervals.

We found several song modifications made by European robins in response to the background noise amplitude (song length = 131s—322s). The mean background noise level across all 25 recordings was 58 dB, with a range from 28 dB to 83 dB (see Figure S1 for more details on the sites sampled and their background noise). The full-null model comparison revealed background noise significantly affected syllable rate, syllable duration, phrase duration, minimum frequency, and frequency bandwidth (Table 1). A summary of the full BLMM results is shown in Table 2. Robins sang fewer syllables per phrase (LRT: *χ*^2^ = 14.97, df = 1, p = 0.0001), with longer average duration (LRT: *χ*^2^ = 16.47, df = 1, p < 0.0001), in noisier environments. Additionally, they sang longer phrases (LRT: *χ*^2^ = 4.95, df = 1, p = 0.026), while phrase rate per minute was unaffected (Fig. 2). Each individual phrase recorded was unique within and between individuals.

**Table 1.** Bayesian linear mixed model full-null model comparison results exploring the effect of background noise amplitude on European robin song parameters. AIC indicates the Akaike Information Criterion for the full model of each response variable, *ω*_*i*_ indicates the Akaike weight of the full model over the null model, ∆ AIC indicates the difference in AIC values from the full to the null model (ie., *null AIC—full AIC*). Square root transformed variables and significant results are indicated by † and *, respectively.

Response Variable	AIC	*ω*_*i*_	∆ AIC	LRT (1 d.f.)
Syllables per Phrase	122.2	0.957	13.0	*χ* ^2^ = 14.97, *p* = 0.0001 *
Syllable Duration	−51.1	0.999	0.7	*χ* ^2^ = 16.47, *p* < 0.0001 *
Phrase Rate †	17.1	0.296	−1.7	*χ* ^2^ = 0.27, *p* = 0.603
Phrase Duration	58.3	0.814	3.0	*χ* ^2^ = 4.95, *p* = 0.026 *
Minimum Frequency	365.1	0.990	9.2	*χ* ^2^ = 11.24, *p* = 0.0008 *
Maximum Frequency †	200.2	0.146	−1.6	*χ* ^2^ = 14.97, *p* = 0.566
Frequency Bandwidth †	191.9	0.888	7.6	*χ* ^2^ = 9.60, *p* = 0.002 *

**Table 2.** Summary of the full Bayesian linear mixed model exploring the effect of background noise amplitude on European robin song parameters. The marginal R^2^ reflects the variance explained by background noise, while the conditional R^2^ is the proportion of variance explained by the full model (background noise and location) and the difference between values indicates the variance explained by the location random effects. Square root-transformed variables are indicated by †.

Response Variable	Intercept [SE]	Slope [SE]	Marginal R^2^	Conditional R^2^	t-value
Syllables per Phrase	10.84 [0.81]	−0.143 [0.036]	0.38	0.57	−3.95
Syllable Duration	0.242 [0.033]	0.0058 [0.0011]	0.45	0.74	5.10
Phrase Rate †	3.14 [0.091]	−0.0022 [0.0044]	0.01	0.23	−0.50
Phrase Duration	2.39 [0.25]	0.0285 [0.0103]	0.22	0.51	2.76
Minimum Frequency	2519 [142]	15.1 [4.55]	0.24	0.68	3.33
Maximum Frequency †	26.34 [3.57]	0.076 [0.173]	0.01	0.24	0.44
Frequency Bandwidth †	30.22 [2.84]	0.415 [0.146]	0.26	0.39	2.84

**Fig. 3.**
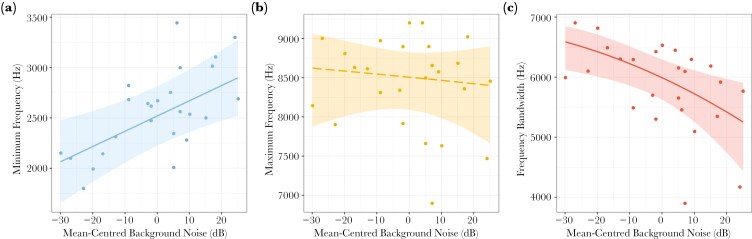
Predicted effect of background noise amplitude on frequency variables in European robin songs: (A) minimum phrase frequency, (B) back-transformed maximum phrase frequency, and (C) back-transformed frequency bandwidth. Significant and non-significant relationships are indicated by solid and dashed lines, respectively. Dots represent mean individual song measures, and the shaded ribbons show the model-derived 0.95 confidence intervals.

Pitch components were also changed with background noise (Fig. 3). Minimum phrase frequency increased with noise amplitude (LRT: *χ*^2^ = 11.24, df = 1, p = 0.0008), although the conditional *R*^2^ was 0.68, much higher than the marginal *R*^2^ of 0.24, suggesting variation in location also influences this parameter (e.g., chronic background noise level effects, habitat differences) (Table 2). Unlike minimum frequency, maximum frequency was unaffected by background noise (LRT: *χ*^2^ = 0.33, df = 1, p = 0.566). The convergence of the phrase frequency extremes reduced the frequency bandwidth (LRT: *χ*^2^ = 9.60, df = 1, p = 0.002).

## Discussion

Winter in the Western Palearctic limits food and shelter while increasing predation risk, making survival dependent on securing territories with sufficient resources and refuge (Cuadrado 1997; Dunn et al. 2003; Brown and Sherry 2006; Johnson et al. 2006; Johnstone 2008). With fewer than half of European robins typically surviving winter (Adriaensen and Dhondt 1990), noise pollution that disrupts song-based territory defense could be detrimental unless mitigated. However, the impact of noise on non-sexually selected, non-breeding songs remains largely understudied—a gap we sought to address. Here, we showed that European robins also adjust their non-breeding songs in response to noise pollution through both frequency and structural modifications.

Animals across diverse taxa often respond to noise pollution by raising the frequency of acoustic signals (Roca et al. 2016a; Kunc and Schmidt 2021). Our findings suggest that European robins increase the non-breeding song minimum frequency from around 2 kHz in the quietest environments to 3 kHz in the noisiest, mirroring the range of experimentally induced increases in the breeding season (McLaughlin and Kunc 2013). Together, these results demonstrate that robins adjust their minimum frequency consistently with noise year-round. A higher minimum frequency helps avoid masking by reducing the signal’s spectral overlap with noise pollution, extending the communication range (Nemeth and Brumm 2009). In noisy environments, higher frequency vocalisations may also be more effective in eliciting behavioral responses in receivers (Halfwerk et al. 2011). The changes in minimum frequency are unlikely to be a result of location variation because, although there was variance explained by the random intercepts, the full model with background noise performed better than one considering location alone. In contrast, the frequency of the highest frequency elements did not change, likely because these are less prone to acoustic masking or because of physical constraints in the syrinx when generating song. As such, robins compress their frequency bandwidth rather than shifting their entire range which reduces frequency complexity (Benedict and Najar 2019). The significant relationship between background noise and transformed bandwidth indicates a strong underlying effect after normalizing its distribution.

In addition to the frequency adjustments, European robins extended phrase duration by singing fewer but longer syllables per phrase in noisy environments. Along with frequency bandwidth, these are commonly used metrics for song complexity, although they are not necessarily concordant making it important to interpret each component independently (Benedict and Najar 2019). By lengthening phrases while maintaining the same number of phrases per minute, robins sing for a greater proportion of time, which may enhance the signal-to-noise ratio and mitigate acoustic masking (Brumm and Slabbekoorn 2005). In contrast, European robins shorten breeding song phrases when experimentally exposed to noise (McLaughlin and Kunc 2013). This difference may reflect the distinct selective pressures shaping breeding and non-breeding songs, as winter songs are simpler and longer than breeding songs (Collar 2020), highlighting an interesting avenue for future research. Furthermore, using fewer, longer syllables may make the song clearer and improve transmission, as complex, fast sequences may be harder to hear with background noise. Alternatively, singers may balance the energetic costs of producing longer syllables and phrases. Producing fewer, longer syllables could allow for efficient communication with reduced energetic cost compared with producing more syllables.

Comprehensive research into how noise pollution affects avian vocalisations is crucial to inform conservation strategies and evaluate the impact of human-induced rapid environmental change on biodiversity. In combination with past work in the breeding season (Montague et al. 2012; McLaughlin and Kunc 2013; McMullen et al. 2014), our findings contribute towards a complete overview of European robin song adjustments within and between breeding seasons. This forms the foundation for researchers to explore how noise impacts an individual’s life history and ecology (Schroeder et al. 2012), and how the vocal adjustments relate to winter survival and territory defense. In addition to the plastic adjustments to background noise, the random intercepts fitted for the six sites also varied for most of the parameters, which could reflect long-term song changes to chronic exposure to noise pollution, whereby individuals learn to sing adjusted songs. This aspect could be explored in future research.

Anthropogenic noise elicits changes in acoustic behavior, which may impair natural communication by affecting the interpretation of a signaler’s fitness and its ability to maintain a territory (Slabbekoorn and Peet 2003; Halfwerk et al. 2011; Roca et al. 2016a; Kunc and Schmidt 2021). These pose additional challenges for wintering European robins. Song plays a central role in territorial defense, and during the resource-scarce non-breeding season survival depends upon access to a quality territory with sufficient nutrients and shelter (Cuadrado 1997; Oosterveld and Frauendorf 2024). For example, although singing at a higher pitch reduces potential masking, higher-frequency sounds attenuate more rapidly over distance (Bradbury and Vehrencamp 1998). Moreover, increasing pitch and reducing complexity likely affect how rivals perceive the singer’s fitness, as lower frequency sounds are often honest indicators of body size and fitness across taxa (Bradbury and Vehrencamp 1998; Hall et al. 2013). Furthermore, in closely related flycatchers, male breeding song complexity reliably reflects brain size and quality (Greig-Smith 1982; Bartsch et al. 2015). These noise-induced adjustments cause high-quality singers to produce songs resembling those of lower-quality individuals, potentially masking true fitness and weakening their chances of winter survival and they may also affect success in the subsequent breeding season (Hoelzel 2008). Because it remains unclear whether these short-term adjustments have evolutionary consequences by enhancing survival (Gross et al. 2010; Moiron et al. 2015), future studies should investigate how vocal plasticity influences winter territory size and quality, predation rates, and individual survival and body condition.

Our findings add to the understanding of how anthropogenic sounds alter song in wild birds, by providing information about the non-breeding season. Although our results align with previous experimental evidence from the same study species (Montague et al. 2012; McLaughlin and Kunc 2013; McMullen et al. 2014), we acknowledge that some limitations may be present. Firstly, the mobile phone application we used to measure background noise amplitude may be less accurate than professional sound meters, although it does provide the advantage of being easily accessible. Using this equipment, significant relations were still identified in the acoustic analysis that concur with established findings across taxa (Kunc and Schmidt 2021). This approach has previously been used in citizen science studies which highlight its value in large-scale urban ecology research (Longo et al., 2020; Zipf et al., 2020), and our findings further support its use. Additionally, we infer a causal effect of background noise on robin songs based on correlation. However, since experimental studies have previously confirmed that noise directly affects multiple song parameters in European robins during the breeding season (Montague et al. 2012; McLaughlin and Kunc 2013; McMullen et al. 2014), it is reasonable to assume this relationship extends to the non-breeding season. Therefore, these limitations should not detract from the validity and reliability of this study and could yet be addressed with future research.

In conclusion, our results contribute to the growing body of evidence that noise pollution affects animal acoustic communication (Ey and Fischer 2009; Roca et al. 2016a; Kunc and Schmidt 2021), and demonstrate that this effect persists during the non-breeding season when song is shaped by natural rather than sexual selection (Catchpole 1987; Adriaensen and Dhondt 1990; Cuadrado 1997). To survive winter, European robins defend territories with adequate resources and shelter (Adriaensen and Dhondt 1990; Cuadrado 1997; Dunn et al. 2003; Brown and Sherry 2006; Johnson et al. 2006; Johnstone 2008). Additionally, greater reproductive success in the breeding season has been linked to frequent singing during autumn and winter (Hoelzel 2008). Therefore, since song plays a crucial role in this process, effectively coping with environmental constraints like noise pollution which impairs song transmission can enhance a robin’s chances of survival. Increasing the minimum frequency while simplifying song structure likely reduces acoustic masking and enhances transmission in noisy environments (Halfwerk et al. 2011; Kunc and Schmidt 2021), and similar effects could be expected in other animal species using acoustic signals to defend non-breeding territory. However, to assess whether the vocal plasticity observed provides adaptive benefits (Moiron et al. 2015), researchers should investigate how these adjustments impact territorial defense, resource acquisition, and ultimately, winter survival. Lastly, these findings can inform future conservation efforts and nature management, as well as planning and legislation for subsequent infrastructure and urban development.

## Supplementary Material

araf070_suppl_Supplementary_Material

## Data Availability

Analyses reported in this article can be reproduced using the data provided by Proudfoot and Norton (2025).
