# Supplementary material for: Non-breeding European robins adjust their song in noisy environments
Source: Behav Ecol. 2025 Jun 12;36(4):araf070. doi: 10.1093/beheco/araf070 (PMC12235003; doi:10.1093/beheco/araf070)
Supplement: araf070_suppl_Supplementary_Material [file araf070_suppl_supplementary_material.docx]

**Supplemental Material**

**Location Descriptions:**

In this study we recorded European robin songs across six sites in the English Midlands. The sites were selected because their primary source of noise pollution was road traffic, and background noise levels varied both within and between sites (Figure S1). Three sites were located in and around Leicester city: Aylestone Meadows Local Nature Reserve, London Road, and Victoria Park. Aylestone Meadows LNR (52°36'37.4"N 1°09'11.7"W) is Leicester’s largest nature reserve at 22-acres, situated on the south of the city and contains open floodplains, woodland, park land and hedgerows. Major roads run along its eastern and southern boundaries. London Road (52°37'05.3"N 1°06'34.9"W) in Leicester is one of the city's major thoroughfares, connecting the city center to the southern and southeastern parts of Leicester. Two recordings were made in a residential area that had a high level of background noise. The last site sampled in Leicester was Victoria Park (52°37'16.5"N 1°07'14.0"W), a large park next to the University of Leicester located within a residential area containing cultivated grassland, lanes lined with mature oak and a small wooded area. It is bounded by London Road to the north and Victoria Park Road to the east which have high levels of road traffic.

In addition to the three sites in Leicester, three sites were sampled in Warwickshire: Lighthorne Village, Oakley Wood, and Warwick Racecourse. Lighthorne Village (52°11'58.0"N 1°30'19.3"W) is a small village with a population of approximately 360 residents surrounded by arable farmland, hedgerows and country lanes. Road traffic is almost exclusively made up of residents and therefore has a low level of noise pollution. The second location sampled in Warwickshire was Oakley Wood (52°14'02.4"N 1°33'11.0"W), a mature mixed woodland with a thick undergrowth. This woodland is near the M40 motorway that connects London to Birmingham, and although the road is not visible from the wood, noise from road traffic is audible in certain locations in this site. Last, we sampled Warwick Racecourse (52°16'56.5"N 1°35'55.5"W), which contains a large pasture land protected for ground nesting songbirds. There are also many hedgerows and trees around this site.


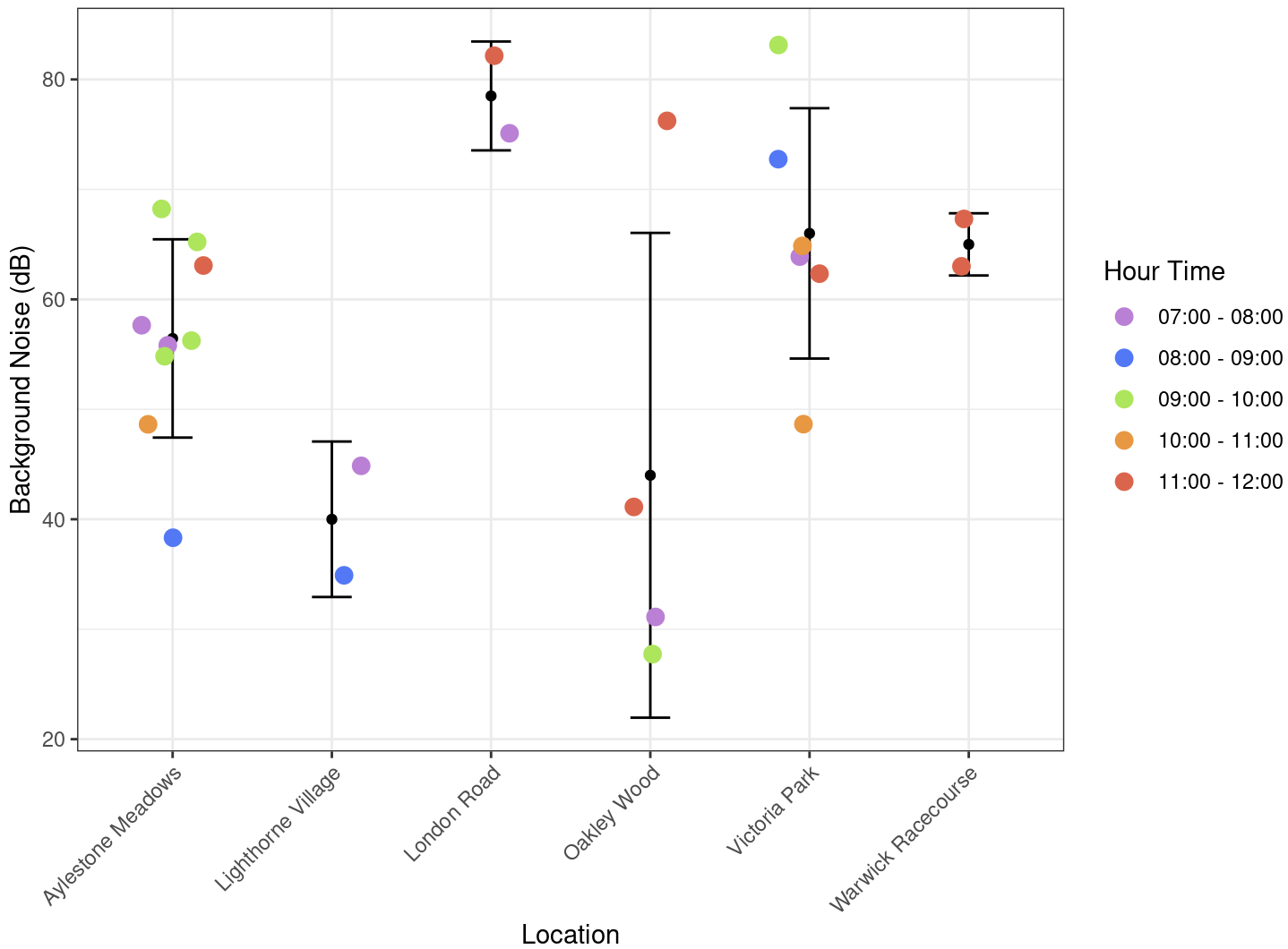


Figure S1. Background noise across locations (Aylestone Meadows, n = 9; Lighthorne Village, n = 2; London Road, n = 2; Oakley Wood, n = 4; Victoria Park, n = 6; Warwick Racecourse, n = 2). Coloured points represent the individual background noise measurements, black points and error bars indicate the mean and standard deviation of background noise per location.


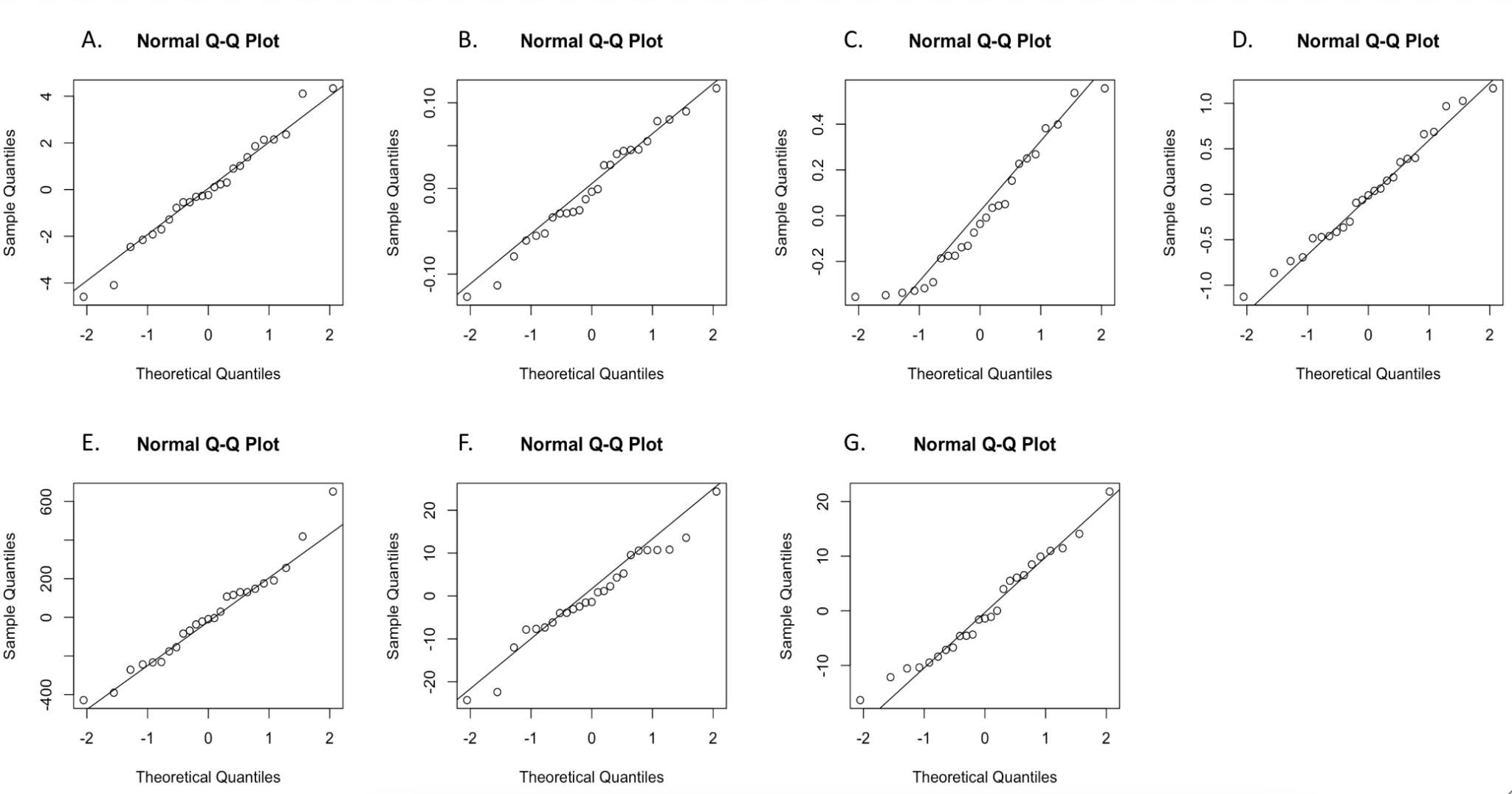


Figure S2. Residual Q-Q plots per parameter model used to validate the assumption of normality: (A) Syllables per phrase, (B) syllable duration, (c) phrase rate (square-root transformed), (D) phrase duration, (E) minimum frequency, (F) maximum frequency (square-root reflection transformed), and (G) frequency bandwidth (square-root reflection transformed).

Table S1. Full model output for syllables per phrase with background noise level as a fixed effect and with location of recording fitted with random intercepts.

| Random effects | | |
| --- | --- | --- |
| Group | Variance | Standard Deviation |
| Location (Intercept) | 2.338 | 1.529 |
| Residual | 5.340 | 2.311 |
| Fixed effects | | |
| Group | Estimate | Standard Error |
| (Intercept) | 10.83584 | 0.81052 |
| Background Noise Level | -0.14285 | 0.03618 |
| Correlation of Fixed Effects (Background Noise Level): -0.006 | | |

Table S2. Full model output for syllable duration with background noise level as a fixed effect and with location of recording fitted with random intercepts.

| Random effects | | |
| --- | --- | --- |
| Group | Variance | Standard Deviation |
| Location (Intercept) | 0.004957 | 0.07041 |
| Residual | 0.004598 | 0.06781 |
| Fixed effects | | |
| Group | Estimate | Standard Error |
| (Intercept) | 0.241655 | 0.032708 |
| Background Noise Level | 0.005814 | 0.001139 |
| Correlation of Fixed Effects (Background Noise Level): -0.009 | | |

Table S3. Full model output for phrase rate with background noise level as a fixed effect and with location of recording fitted with random intercepts.

| Random effects | | |
| --- | --- | --- |
| Group | Variance | Standard Deviation |
| Location (Intercept) | 1.002 | 1.001 |
| Residual | 3.639 | 1.908 |
| Fixed effects | | |
| Group | Estimate | Standard Error |
| (Intercept) | 9.96729 | 0.58525 |
| Background Noise Level | -0.01479 | 0.02880 |
| Correlation of Fixed Effects (Background Noise Level): -0.004 | | |

Table S4. Full model output for phrase duration with background noise level as a fixed effect and with location of recording fitted with random intercepts.

| Random effects | | |
| --- | --- | --- |
| Group | Variance | Standard Deviation |
| Location (Intercept) | 0.2444 | 0.4944 |
| Residual | 0.4145 | 0.6439 |
| Fixed effects | | |
| Group | Estimate | Standard Error |
| (Intercept) | 2.38954 | 0.24886 |
| Background Noise Level | 0.02849 | 0.01033 |
| Correlation of Fixed Effects (Background Noise Level): -0.007 | | |

Table S5. Full model output for minimum frequency with background noise level as a fixed effect and with location of recording fitted with random intercepts.

| Random effects | | |
| --- | --- | --- |
| Group | Variance | Standard Deviation |
| Location (Intercept) | 98785 | 314.3 |
| Residual | 70876 | 266.2 |
| Fixed effects | | |
| Group | Estimate | Standard Error |
| (Intercept) | 2518.596 | 142.353 |
| Background Noise Level | 15.144 | 4.553 |
| Correlation of Fixed Effects (Background Noise Level): -0.009 | | |

Table S6. Full model output for transformed maximum frequency with background noise level as a fixed effect and with location of recording fitted with random intercepts.

| Random effects | | |
| --- | --- | --- |
| Group | Variance | Standard Deviation |
| Location (Intercept) | 38.63 | 6.215 |
| Residual | 129.78 | 11.392 |
| Fixed effects | | |
| Group | Estimate | Standard Error |
| (Intercept) | 26.34131 | 3.56925 |
| Background Noise Level | 0.07631 | 0.17303 |
| Correlation of Fixed Effects (Background Noise Level): -0.005 | | |

Table S7. Full model output for transformed frequency bandwidth with background noise level as a fixed effect and with location of recording fitted with random intercepts.

| Random effects | | |
| --- | --- | --- |
| Group | Variance | Standard Deviation |
| Location (Intercept) | 20.8 | 4.561 |
| Residual | 96.8 | 9.839 |
| Fixed effects | | |
| Group | Estimate | Standard Error |
| (Intercept) | 30.2235 | 2.8360 |
| Background Noise Level | 0.4149 | 0.1459 |
| Correlation of Fixed Effects (Background Noise Level): -0.003 | | |
